# Predicting the prognosis of glioma patients with TERT promoter mutations and guiding the specific immune profile of immune checkpoint blockade therapy

**DOI:** 10.18632/aging.205668

**Published:** 2024-03-18

**Authors:** Wenpeng Cao, Jinzhi Lan, Chujiao Hu, Jinping Kong, Limin Xiang, Zhixue Zhang, Yating Sun, Zhirui Zeng, Shan Lei

**Affiliations:** 1 Department of Anatomy, School of Basic Medicine, Guizhou Medical University, Guiyang, Guizhou 550025, China; 2 Key Laboratory of Human Brain Bank for Functions and Diseases of Department of Education of Guizhou, Guizhou Medical University, Guiyang, Guizhou 550025, China; 3 Center for Tissue Engineering and Stem Cell Research, Guizhou Medical University, Guiyang, Guizhou 550004, China; 4 Department of Physiology, School of Basic Medicine, Guizhou Medical University, Guiyang, Guizhou 550025, China; 5 State Key Laboratory of Functions and Applications of Medicinal Plants, Guizhou Medical University, Guiyang, Guizhou 550025, China; 6 Guizhou Provincial Engineering Technology Research Center for Chemical Drug R&D, Guiyang, Guizhou 550025, China

**Keywords:** immunity, risk model, glioma, TERT promoter mutation, immune checkpoint blockade therapy

## Abstract

The telomerase reverse transcriptase promoter (TERTp) is frequently mutated in gliomas. This study sought to identify immune biomarkers of gliomas with TERTp mutations. Data from TCGA were used to identify and validate survival-associated gene signatures, and immune and stromal scores were calculated using the ESTIMATE algorithm. High stromal or immune scores in patients with TERTp-mutant gliomas correlated with shorter overall survival compared to cases with low stromal or immune scores. Among TERTp-mutant gliomas with both high immune and high stromal scores, 213 commonly shared DEGs were identified. Among 71 interacting DEGs representing candidate hub genes in a PPI network, HOXC6, WT1, CD70, and OTP showed significant ability in establishing subgroups of high- and low-risk patients. A risk model based on these 4 genes showed strong prognostic potential for gliomas with mutated TERTp, but was inapplicable for TERTp-wild-type gliomas. TERTp-mutant gliomas with high-risk scores displayed a greater percentage of naïve B cells, plasma cells, naïve CD4 T cells, and activated mast cells than low-risk score gliomas. TIDE analysis indicated that immune checkpoint blockade (ICB) therapy may benefit glioma patients with TERTp mutations. The present risk model can help predict prognosis of glioma patients with TERTp mutations and aid ICB treatment options.

## INTRODUCTION

Among all intracranial malignant neoplasms, gliomas are the most common and aggressive forms of primary brain tumors [1, 2]. The poor survival outcome of glioma patients is related to the limited efficacy of current treatment options, including surgery, radiotherapy, and chemotherapy [3]. Multiple genetic anomalies are commonly present in gliomas, including IDH mutations [4], 1p/19q co-deletion [5], and TElomerase Reverse Transcriptase promoter (TERTp) mutations [6]. TERTp mutations are among the most common somatic non-coding mutations in human cancers, driving cancer cell immortalization and tumor progression by reactivating telomerase activity [7, 8]. Indeed, TERTp mutations occur in more than half of all glioma grades, and in over 80% of WHO grade IV gliomas (i.e., glioblastoma, GBM), the most lethal glioma type [9]. While the prognostic significance of TERTp mutations is still equivocal and may depend on concurrent mutations [10], there is an urgent interest in developing therapeutic strategies to inhibit TERT activity in cancer cells [11]. TERTp mutations are associated with poor survival in patients with gliomas [12], and thus different treatment strategies may be needed for patients with wild-type and mutant TERTp. Therefore, the identification of biomarkers associated with TERTp mutations may be helpful to guide glioma treatment.

In the current study, the TCGA database was searched to identify biomarkers associated with TERTp mutations in patients with gliomas. After analysis of immune and stromal cell distributions in TERTp-mutant gliomas, bioinformatics methods and COX regression techniques were applied to identify differentially regulated genes associated with survival. From these data, a risk model based on 4 immune-related genes was developed and applied to differentiate low-risk from high-risk patients and to construct a nomogram to predict overall survival. The present risk model will hopefully serve to guide the treatment of gliomas containing mutations in the TERT promoter.

## RESULTS

### High stromal/immune scores predict lower overall survival in glioma patients with TERTp mutations

**Figure 1:**
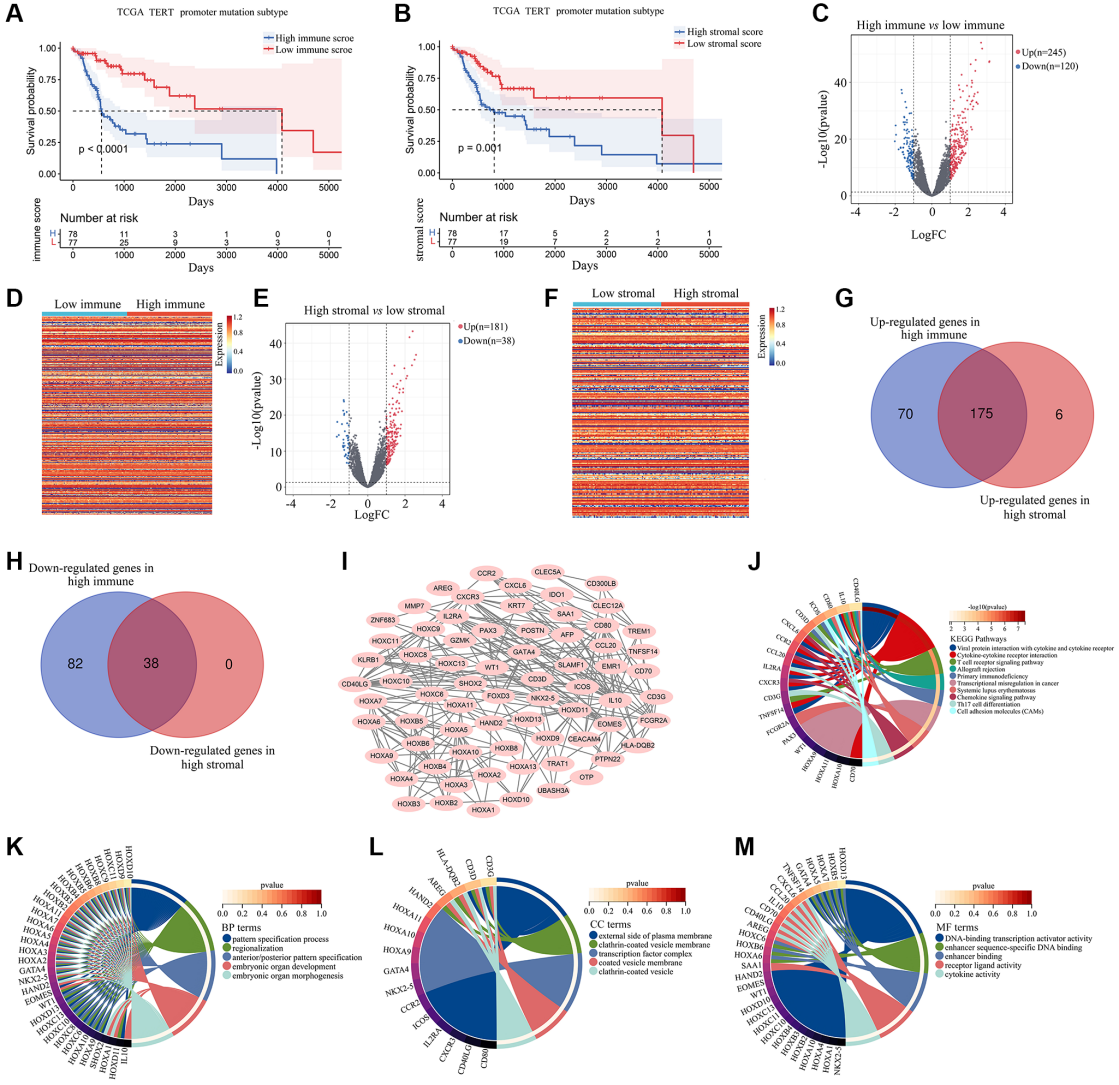
**Effect of stromal and immune scores on survival of patients with TERTp-mutant gliomas.** (**A**) Kaplan-Meier survival analysis of patients in the high and low immune score groups. (**B**) Kaplan-Meier survival analysis of patients in the high and low stromal score groups. (**C**) Volcano plot showing differential gene expression for the high and low immune groups. (**D**) Heat map depicting differential gene expression between TERTp-mutant gliomas in the high and low immune groups. (**E**) Volcano plot showing differential gene expression for TERTp-mutant gliomas in the high and low immune groups. (**F**) Heat map showing DEGs between TERTp-mutant gliomas in the high and low stromal groups. (**G**) Venn diagram of upregulated genes in the high stromal and immune groups. (**H**) Venn diagram of downregulated genes in the high stromal and immune groups. (**I**) PPI network constructed using 71 overlapping DEGs while removing isolated genes. Genes in the PPI network was set as candidate hub genes. (**J**) Kyoto Encyclopedia of Genes and Genomes (KEGG) analysis of candidate hub genes. (**K**) GO-Biological process (BP) analysis of candidate hub genes. (**L**) GO-Cellular component (CC) analysis of candidate hub genes. (**M**) GO-Molecular function (MF) analysis of candidate hub genes.

After retrieving gene expression profiles from glioma patients with TERTp mutations in TCGA, we evaluated the association between immune and stromal scores, obtained with the ESTIMATE algorithm, and overall survival (OS). A lower OS rate was detected after Kaplan-Meier survival analysis for gliomas with high immune (Figure 1A) and stromal (Figure 1B) scores. We next conducted comparative gene expression analysis between the high and low immune/stromal groups. A total of 245 genes were upregulated, whereas 120 genes were downregulated, in TERTp-mutated glioma tissues from the high, compared to the low, immune group (Figure 1C, 1D). In turn, 181 upregulated genes and 38 downregulated genes were detected in glioma tissues in the high, compared to the low, stromal group (Figure 1E, 1F). Upon comparative analysis of TERTp-mutant glioma tissues with high stromal and high immune scores, a total of 213 DEGs (175 co-upregulated and 38 co-downregulated ones; Figure 1G, 1H) were found to be shared among the two groups (Supplementary Table 1).

After constructing a PPI network, we identified 71 genes involved in gene-to-gene interactions (Figure 1I). Genes related to stromal and immune scoring in TERTp-mutant gliomas were identified as candidate hub genes. KEGG enrichment analysis revealed that these candidate hub genes interact with cytokines and cytokine receptors, showing enrichment in ‘cytokine-cytokine receptor interaction’, ‘T cell receptor signaling pathway’, ‘allograft rejection’, ‘primary immunodeficiency’, ‘transcriptional misregulation in cancer’, ‘systemic lupus erythematosus’, ‘chemokine signaling pathway’, and ‘Th17 cell differentiation’ (Figure 1J). Gene Ontology (GO)-BP enrichment analysis based on cell adhesion molecule ontology revealed that these candidate hub genes are enriched in pattern specification processes, regionalization, and anterior/posterior pattern specification (Figure 1K). GO-CC enrichment analysis showed enrichment on the external surface of plasma membrane for these candidate hub genes (Figure 1L). Based on GO-MF enrichment analysis, candidate hub genes are enriched in DNA-binding transcription activator activity, enhancer sequence-specific DNA binding, enhancer binding, receptor ligand activity, and cytokine activity (Figure 1M).

### Construction of an immune signature for glioma patients with TERTp mutations

**Figure 2:**
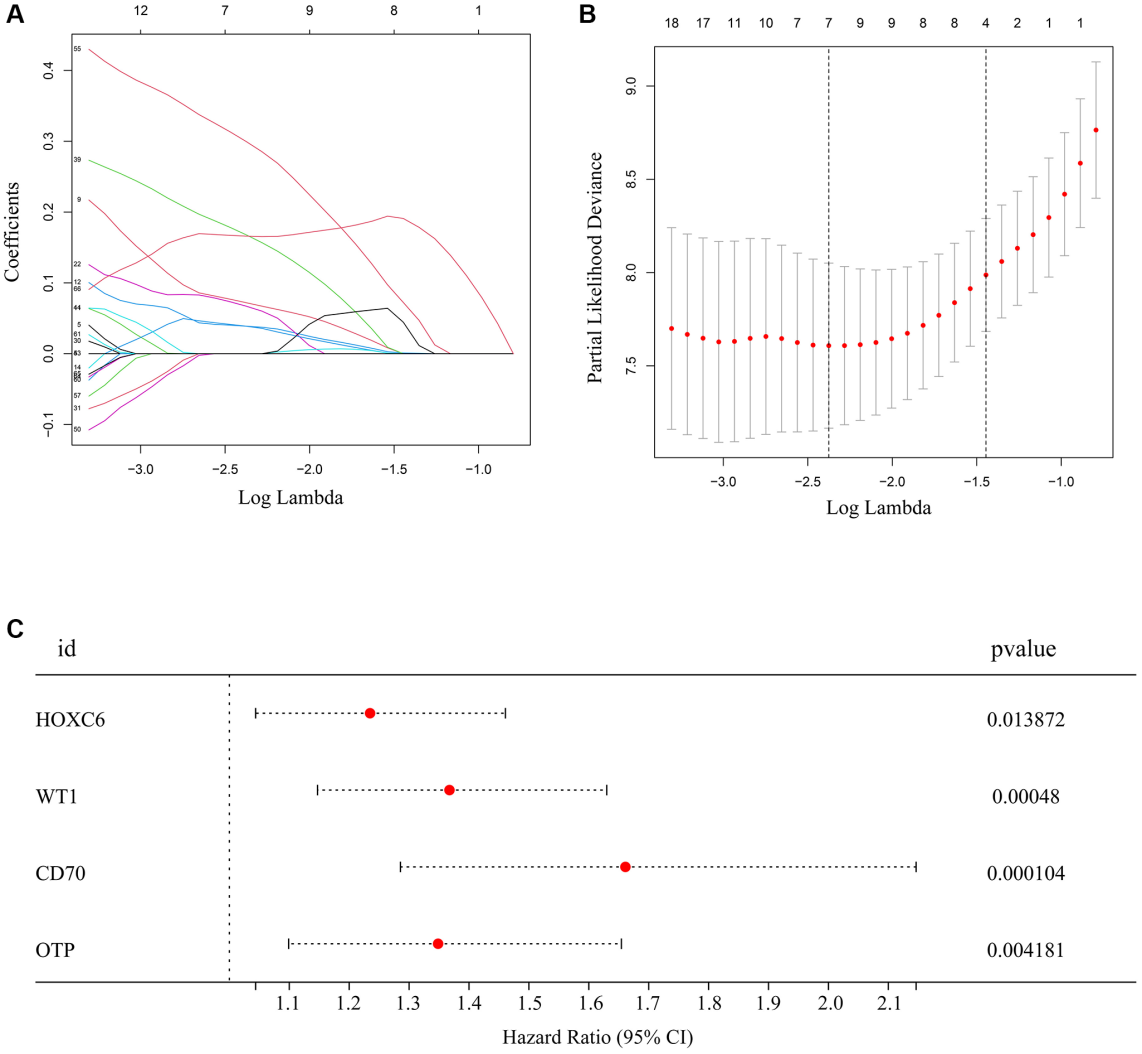
**Hub genes selected to construct the risk model.** (**A**, **B**) LASSO analysis for hub genes associated with the survival rate of glioma patients with TERTp mutations. (**C**) Multivariate Cox regression analysis of HOXC6, WT1, CD70, and OTP. These four genes were used to construct the risk model.

Next, we assessed the association between high immune/high stromal score-related DEGs identified in TCGA-glioma patients with TERTp mutations and survival data. Based on univariate Cox regression, 73 of 213 DEGs were associated with survival (Supplementary Table 2). Upon further analysis using LASSO and Cox regression analyses, seven additional DEGs, including HOXC6, HOXA10, EOMES, WT1, HOXC10, CD70, OTP retained significant associations with overall survival (Figure 2A, 2B). Based on the above analysis, a risk model associated with mutant TERTp in glioma was constructed using gene expression data for HOXC6, WT1, CD70, and OTP, as well as survival data from the TCGA. For each patient, the risk score was calculated as: HOXC6 × 0.211 + WT1 × 0.312 + CD70 × 0.511 + OTP × 0.299 (Figure 2C).

### Risk model validation

**Figure 3:**
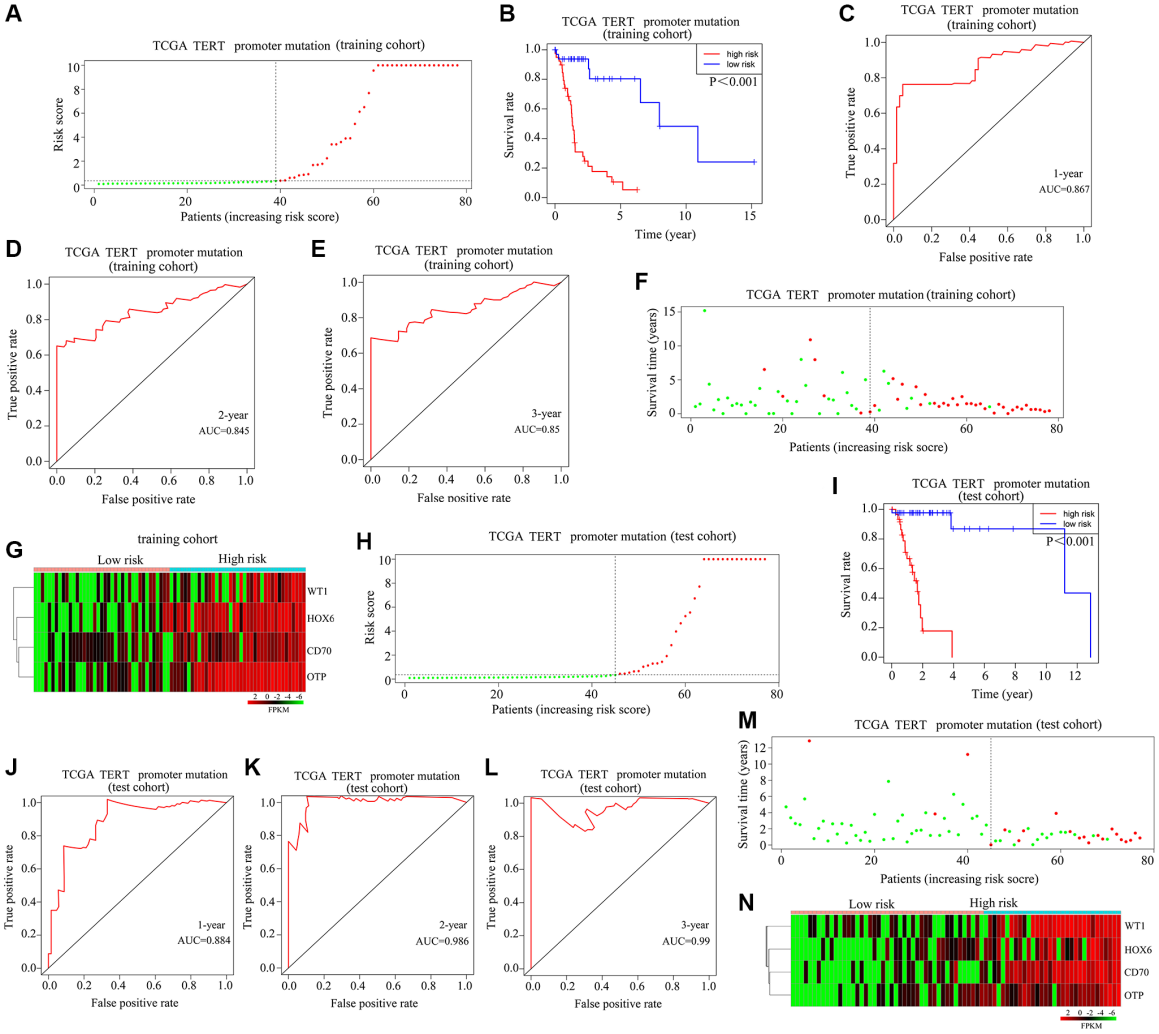
**Validation of the applicability of the risk model in patients with TERTp-mutant gliomas.** (**A**) Patients with TERTp-mutant gliomas in the training cohort were divided into high-risk and low-risk groups based on the median risk score. (**B**) Survival differences between patients in the high- and low-risk scoring groups in the training cohort. (**C**–**E**) Diagnostic value of risk models for 1-year, 3-year, and 5-year survival in the training cohort. (**F**) Survival time as a function of risk score for patients in the training cohort. Green dots represent live cases, and red dots represent dead cases. (**G**) Heatmap depicting expression levels of HOXC6, WT1, CD70, and OTP in glioma samples in the high-risk and low-risk score groups in the training cohort. (**H**) Patients with TERTp-mutant gliomas in the test cohort were divided into high-risk and low-risk groups based on the median risk score. (**I**) Survival differences between patients in the high- and low-risk score groups in the test cohort. (**J**–**L**) Diagnostic value of risk models for 1-year, 3-year, and 5-year survival for patients in the test cohort. (**M**) Survival time as a function of risk score for patients in the high-risk and low-risk scoring groups in the test cohort. Green dots represent living cases, and red dots represent dead cases. (**N**) Heatmap depicting expression levels of HOXC6, WT1, CD70, and OTP in glioma patients in the high-risk and low-risk scoring groups of the test cohort.

To validate the applicability of the risk model described above, glioma patients with TERTp mutations were divided into high-risk and low-risk groups in a training cohort (Figure 3A). In the latter, high-risk scores were associated with lower OS rates (Figure 3B). After ROC analysis, the AUCs for predicting survival one year, three years, and five years after diagnosis were 0.867, 0.845, and 0.85, respectively (Figure 3C–3E). Further analysis indicated a higher mortality risk for patients with high-risk scores (Figure 3F). As shown in Figure 3G, HOXC6, WT1, CD70, and OTP were highly expressed in high-risk vs. low-risk glioma samples. A prognostic risk model and a moderate risk score constructed in the TCGA test cohort were used to categorize glioma cases as high-risk or low-risk (Figure 3H). In this test cohort, the OS rate of glioma patients with high-risk scores was also shorter (Figure 3I). According to ROC curves, AUCs of 0.884, 0.986, and 0.99 were respectively obtained for 1-year, 3-year, and 5-year OS (Figure 3J–3L). It was also observed in the test cohort that patients with high-risk scores died more frequently (Figure 3M). As shown in Figure 3N, HOXC6, WT1, CD70, and OTP expression was upregulated in glioma tissues from patients with high-risk scores. This evidence suggests that the proposed risk model is useful for predicting the prognosis of glioma patients with TERTp mutations.

### Applicability of the risk model in TCGA-glioma patients with wild-type TERTp

**Figure 4:**
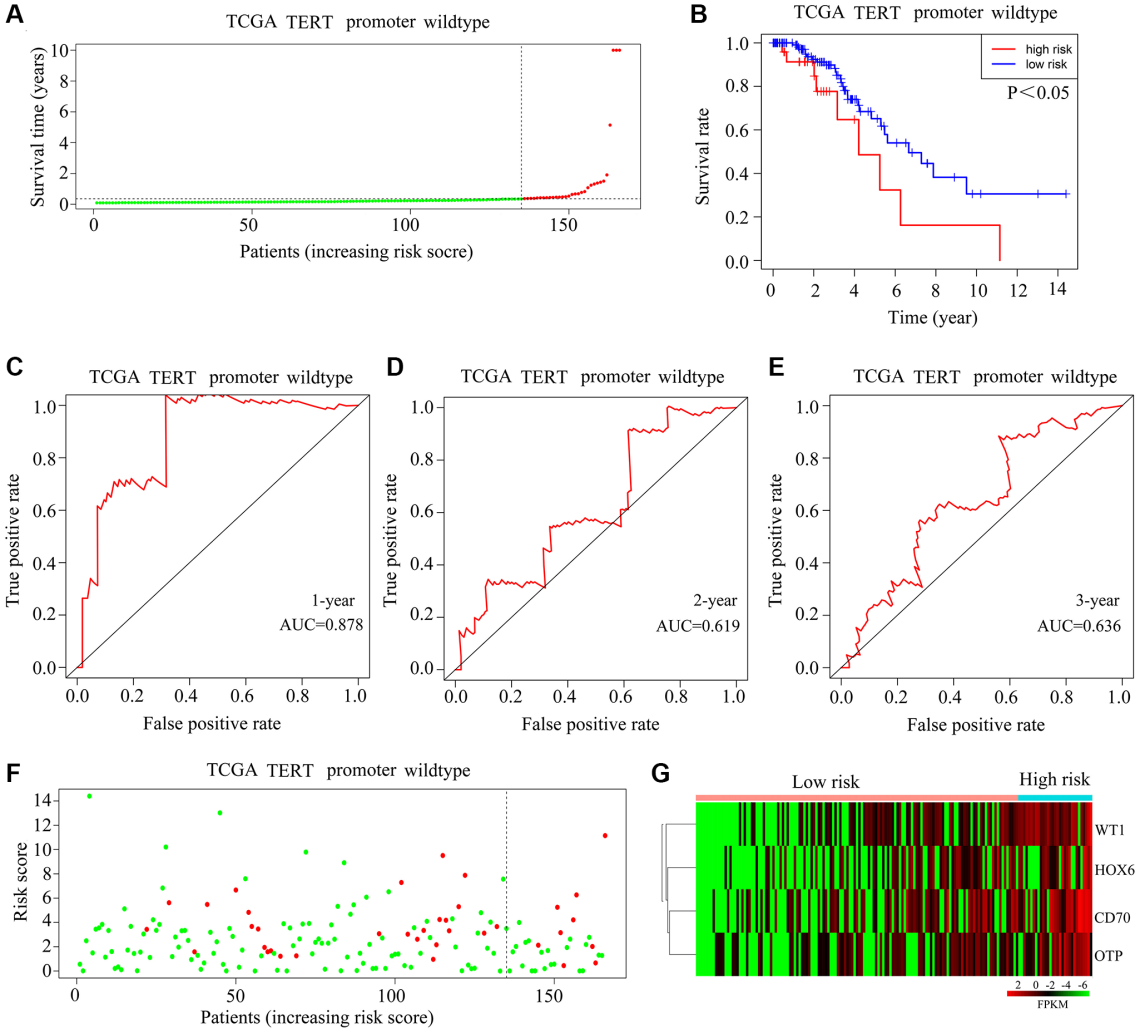
**Validation of the applicability of the risk model in TCGA-glioma patients with wild-type TERTp.** (**A**) Patients with TERTp-wild-type glioma in the TCGA database were divided into high-risk and low-risk groups based on the median risk score. (**B**) Survival differences between patients in the high-risk and low-risk groups. (**C**–**E**) Diagnostic value of risk models for 1-year, 3-year, and 5-year survival rates. (**F**) Survival time as a function of risk score for glioma patients with wild-type TERTp in the high-risk and low-risk groups. Green dots represent living cases, and red dots represent dead cases. (**G**) Heatmap depicting expression levels of HOXC6, WT1, CD70, and OTP in gliomas from patients with wild-type TERTp in the high-risk and low-risk groups.

In the TCGA-glioma cohort, high-risk and low-risk TERTp-wild-type gliomas were classified based on median risk scores (Figure 4A). Survival analysis indicated that patients with high-risk scores lived shorter than those with low-risk scores (Figure 4B). On ROC analysis, AUCs of 0.818, 0.619, and 0.636, predictive, respectively, of 1-year, 3-year, and 5-year survival, were recorded for glioma patients with wild-type TERTp (Figure 4C–4E). There was no significant difference in mortality between high-risk patients and those at low risk (Figure 4F). HOXC6, WT1, CD70, and OTP expression was elevated in glioma tissues from high-risk score patients carrying wild-type TERTp (Figure 4G). However, in patients with wild-type TERTp-wild-type gliomas, the HOXC6-WT1-CD70-OTP risk models did not predict survival, and may thus be specific to glioma cases with TERTp mutations.

### A nomogram based on a 4-gene immune signature has prognostic ability in glioma patients with TERTp mutations

**Figure 5:**
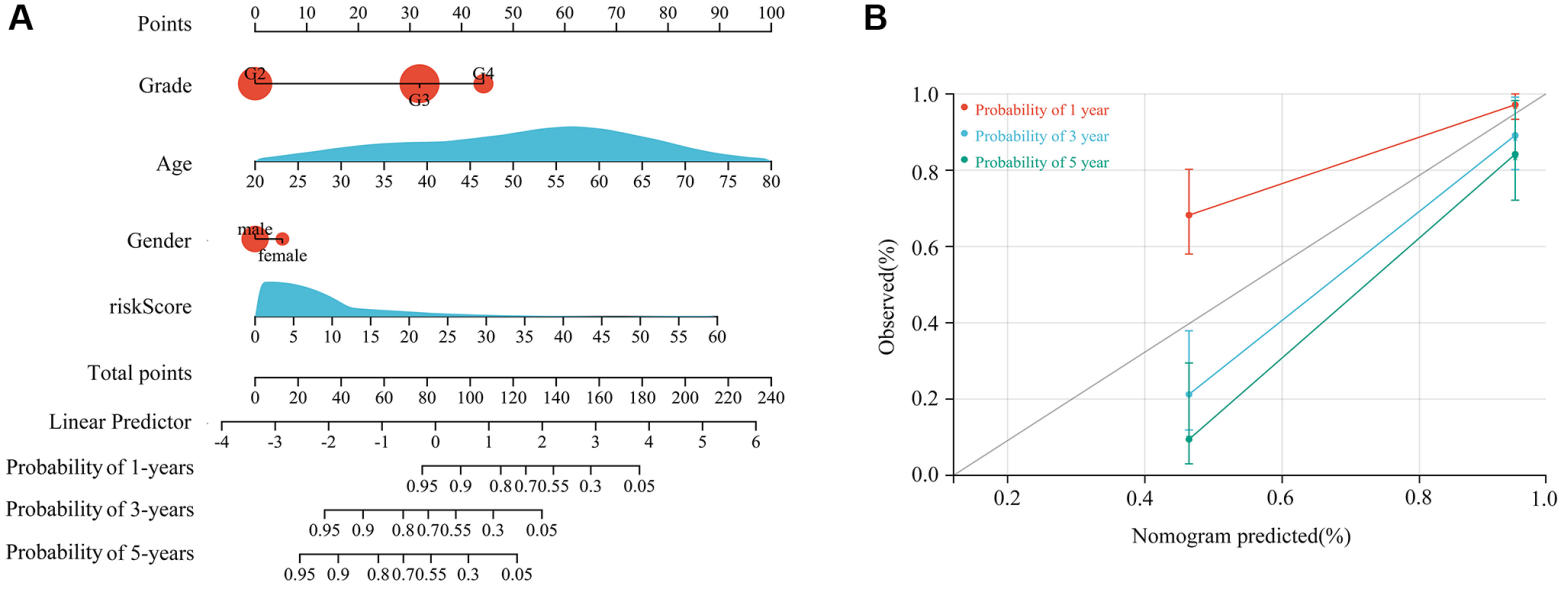
**Construction of a nomogram based on a 4-gene signature risk score and clinical characteristics.** (**A**) Proposed nomogram, incorporating age, gender, glioma grade, and risk score. (**B**) Efficiency of the nomogram in predicting 1-year, 3-year, and 5-year survival for patients with TERTp-mutant gliomas.

Based on multivariate Cox regression analysis, the immune signature created using HOXC6, WT1, CD70, and OTP represented an independent prognostic factor for glioma patients with TERTp mutations. This is evidenced in a column line plot based on signature risk scores and clinical features (Figure 5A), which showed excellent prognostic value for 1-, 3-, and 5-year survival (Figure 5B).

### Analysis of tumor-infiltrating immune cell types and predicted response to immune checkpoint blockade therapy in glioma patients with TERTp mutations

**Figure 6:**
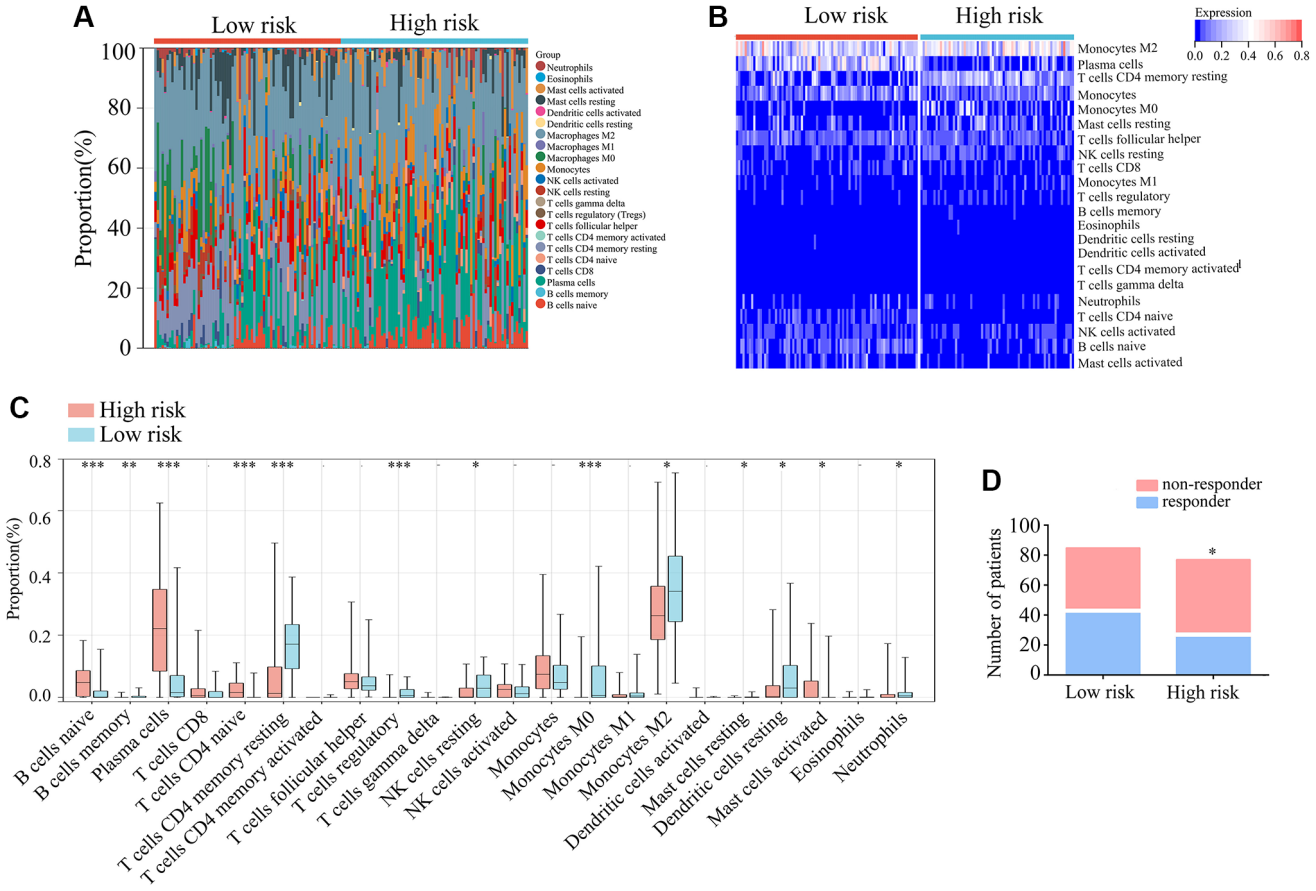
**Immunological characteristics of TERTp-mutant gliomas in TCGA.** (**A**, **B**) Expression matrices showing proportions and expression patterns of 22 tumor-infiltrating immune cell types in TCGA-glioma tissues from patients with TERTp mutations in the high- and low-risk groups. (**C**) Profiling of tumor-infiltrating immune cells in TERTp-mutations-type glioma tissues in the high- and low-risk groups in TCGA. (**D**) Predicted proportions of ICB responders and non-responders among glioma patients with TERTp mutations in the high-risk and low-risk groups.

Research has demonstrated that immune cells infiltrating TERTp-mutant gliomas play a significant role in disease progression. Thus, CIBERSORT was used to analyze the distribution of 22 infiltrating immune cell types in high-risk and low-risk glioma patients with TERTp mutations (Figure 6A, 6B). Compared to low-risk gliomas, high-risk gliomas showed higher proportions of naïve B-cells, plasma cells, naïve CD4 T-cells, and activated mast cells, and lower proportions of memory B cells, resting memory CD4 T cells, regulatory T cells, resting NK cells, M0 and M2 macrophages, dendritic cells, and neutrophils (Figure 6C). In addition, TIDE analysis indicated that immune checkpoint blockade (ICB) may be an effective treatment option for glioma patients with TERTp mutations in the high-risk group (Figure 6D). Hence, we propose that glioma patients with TERTp mutations may benefit from the present risk model to guide their clinical treatment.

### Validation of HOXC6, WT1, CD70, and OTP expression trends in glioma tissues with TERTp mutations

**Figure 7:**
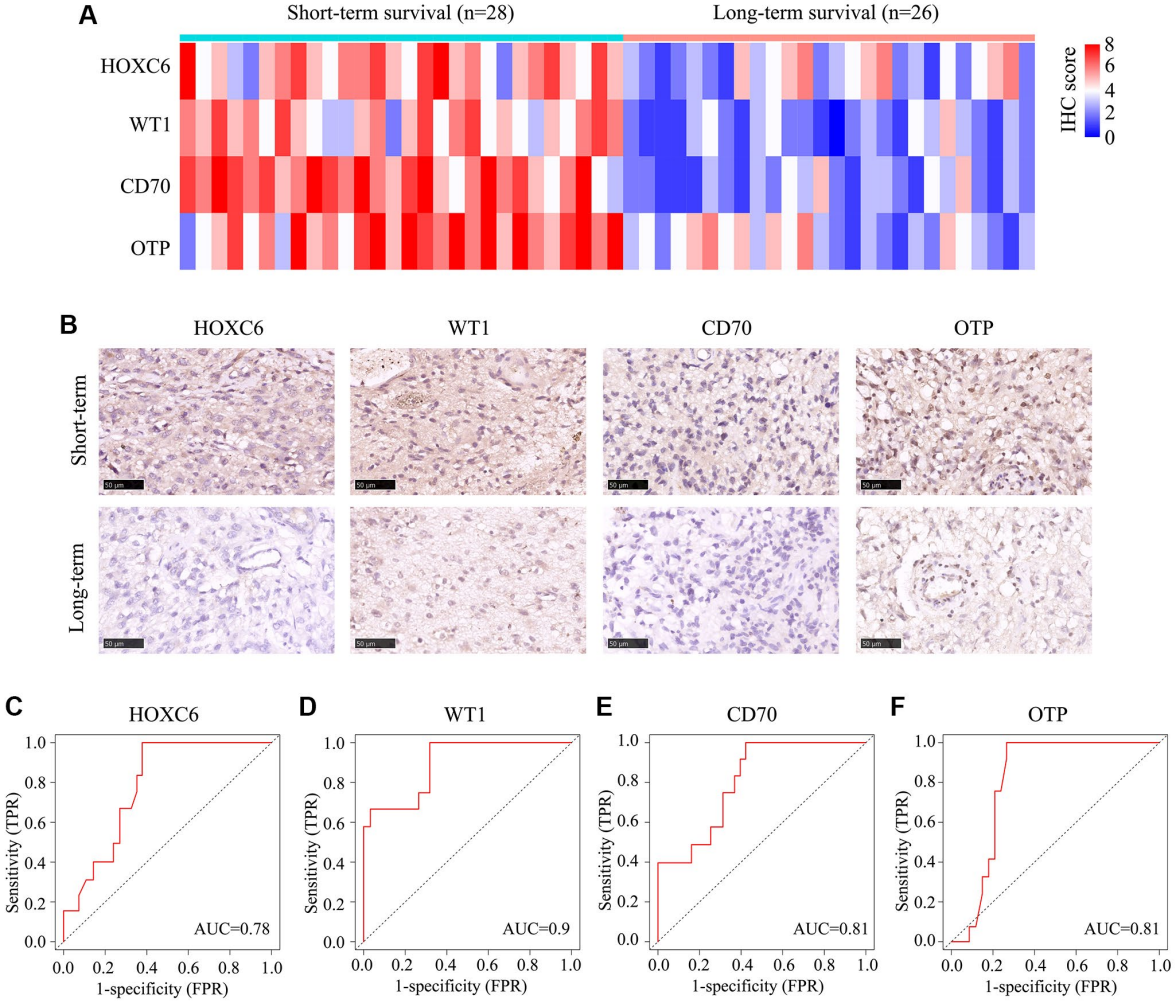
**Expression of HOXC6, WT1, CD70, and OTP in 54 glioma cases with TERTp mutations.** TERTp-mutant glioma samples from 54 glioma patients admitted to our hospital were divided into long- and short-term survival groups based on a survival cut-off of 15 months. (**A**) IHC scores for HOXC6, WT1, CD70, and OTP expression in glioma samples from patients in the long- and short-term survival groups. (**B**) Representative IHC images showing the expression of HOXC6, WT1, CD70, and OTP in glioma samples from patients in the long- and short-term survival groups. (**C**–**F**) Diagnostic value of HOXC6, WT1, CD70, and OTP for distinguishing long- and short-term survivors among glioma patients with TERTp mutations.

We next analyzed 54 glioma samples with TERTp mutations obtained in our institution, divided into long-term and short-term survival groups. Immunohistochemistry revealed higher expression levels of HOXC6, WT1, CD70, and OTP in the long-term vs. the short-term survival group (Figure 7A, 7B). ROC analysis was subsequently applied to determine the significance of HOXC6, WT1, CD70, and OTP expression levels on patient survival. Results indicated a significant prognostic value for the four genes (AUC = 0.78, 0.09, 0.81, and 0.81, respectively) (Figure 7C–7F).

### 5-fluorouracil and gemcitabine may benefit high-risk glioma patients with TERTp mutations

**Figure 8:**
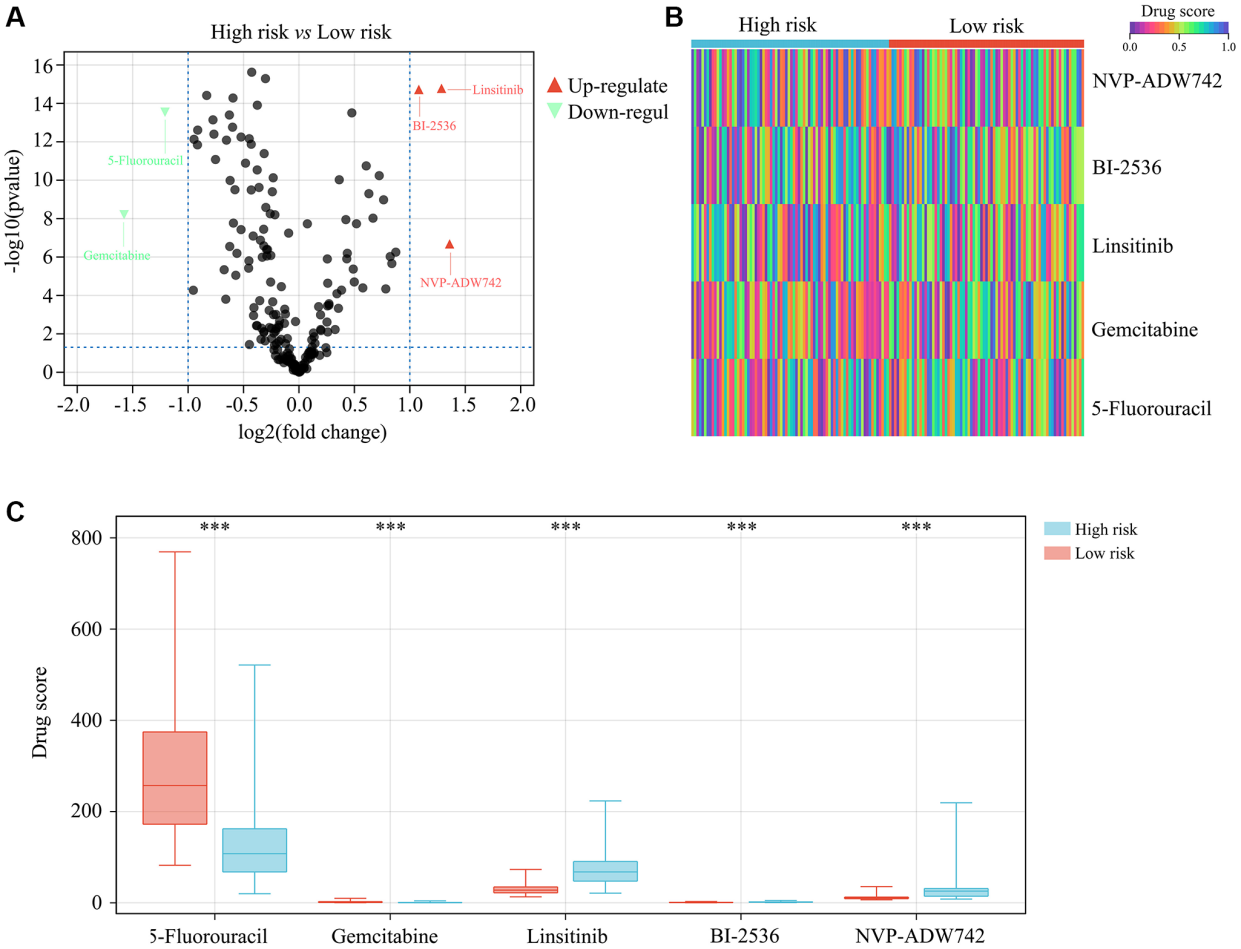
**Drug sensitivity analysis for high-risk TERTp-mutant gliomas.** (**A**–**C**) OncoPredict analysis of drug sensitivity of TERTp-mutant gliomas. Results suggest higher sensitivity to 5-fluorouracil and gemcitabine therapies for glioma patients in the high- vs. the low-risk group. ^***^P < 0.001.

In order to determine which oncology drugs might be appropriate for high-risk TERTp-mutant glioma patients, we integrated the corresponding gene expression profiles into a drug sensitivity matrix using the OncoPredict algorithm. Among 198 candidate drugs, higher sensitivity was predicted for 5-fluorouracil and gemcitabine for this subgroup of patients (Figure 8A–8C).

## DISCUSSION

There is an urgent need for reliable prognostic models for patients with glioblastoma (GBM), the most common and aggressive primary brain tumor. Dysregulation of the immune microenvironment contributes to the progression of gliomas with TERTp mutations [13, 14]. In this work, our analysis of the TCGA-glioma patient cohort exhibiting TERTp mutations showed that patients with high stromal/immune scores had lower survival rates than those with low stromal/immune scores. Upon identification of DEGs between high and low stromal/immune score groups, 73 out of 213 DEGs shared between the high stromal and high immune score groups were obviously correlated with prognosis. In turn, PPI network analysis revealed significant interactions (and prominent enrichment in cytokine signaling pathways) for 71 out of the 213 common DEGs. After LASSO and Cox regression analysis, immune profiles were established based on HOXC6, WT1, CD70, and OTP expression levels. Focusing on the TERTp-mutant glioma subtype, we developed an immune-related gene signature including HOXC6, WT1, CD70, and OTP that showed significant prognostic value for TERTp-mutant gliomas, but not for TERTp wild-type gliomas, in two TCGA cohorts. We thus suggest that TERTp-mutant gliomas may be appropriately assessed based on the present risk model.

Homeobox genes, mainly represented by the HOX gene family, act as critical developmental regulators by influencing cell proliferation, migration, differentiation, and death during axial patterning [15, 16]. HOXC6 was found to be overexpressed in clinical glioma samples, and its knockdown stimulated the WIF-1/Wnt signaling pathway and induced cell cycle arrest and apoptosis in U87 glioma cells [17]. HOXC6 was also found to regulate EMT signaling, and was proposed as a new immunotherapeutic target for gliomas [18, 19]. The orthopedia homeobox (OTP) gene, a member of the homeodomain (HD) family, plays an essential role in development and cell-fate specification of the hypothalamic neuroendocrine system in vertebrates [20]. OTP was identified as a reliable prognostic indicator in lung carcinomas, including neuroendocrine ones [21, 22]. CD70 is a costimulatory molecule involved in T-cell-mediated immunity that critically contributes to recurrent GBM aggressiveness and maintenance [23, 24]. Jin et al. reported that CD70-specific CAR T cells recognize primary CD70+ GBM tumors *in vitro* and mediate the regression of established GBM in xenograft and syngeneic rodent models [25]. Initially identified as a tumor suppressor, the Wilms’ tumor 1 (WT1) gene has been shown to display significant increases in expression across a range of human cancers, including lung and pancreatic cancer [26, 27]. In turn, high levels of WT1 mRNA have been reported in gliomas at advanced clinical stages and with poor prognoses [28, 29].

The tumor microenvironment (TME) consists of different types of cells, including cancer cells, immune/inflammatory cells, vascular cells, and cancer-associated fibroblasts [30]. The progression of gliomas is influenced by immune cells that infiltrate the TME [31]. Glioma-associated macrophages predominantly exhibit the M2 phenotype, which induces angiogenesis and thus enhances tumor aggressiveness. We found that higher levels of naïve B cells, plasma cells, naïve CD4 T cells, and activated mast cells were characteristic of TERTp-mutant glioma patients with high-risk scores in both TCGA cohorts, in association with a lower survival rate. This evidence would suggest an immunosuppressive TME in TERTp-mutant glioma patients with high-risk scores.

Some patients with diverse cancer types, including bladder and lung cancer, have experienced significant benefits from ICB therapies [32, 33]. In these approaches, cancer cells can be killed by blocking immune checkpoints to reactivate deactivated immune cells [34]. There is, however, limited evidence that ICB is effective in gliomas with TERTp mutations. Our TIDE analysis indicated that ICB may improve the prognosis of TERTp-mutant glioma patients in the high-risk score group. Through further OncoPredict analysis, four candidate drugs, among them 5-fluorouracil and gemcitabine, were identified as potentially effective in this group. This evidence may contribute to guiding chemotherapy and targeted therapies for high-risk glioma patients with TERTp mutations.

In conclusion, an immune signature based on HOXC6, WT1, CD70, and OTP expression was shown to serve as an independent and specific prognostic indicator for patients with TERTp- mutant gliomas. Interestingly, the high-risk population classified by this signature was predicted to benefit from ICB. This novel 4-gene, immune-related signature might thus be valuable to guide the treatment of gliomas with TERTp mutations.

## MATERIALS AND METHODS

### Gene expression profiling and estimation of immune and stromal scores

The TCGA genome database was accessed to obtain gene expression profiles from glioma patients. Several probe names were annotated as gene names, and raw gene expression profiles were normalized and centralized. Cases with mutations in the TERTp and those without survival data were excluded from the analysis. In the TCGA cohort, 155 patients had TERTp mutations and 166 patients were TERTp-wild-type. The ESTIMATE R tool was used to calculate immune and stromal scores in glioma tissues harboring TERTp mutations.

### Analysis of differentially expressed genes

A significance threshold of *P* < 0.05 and |logFold-Change | <1 was set to analyze differentially expressed genes (DEGs). Volcano plots were created to visualize and analyze gene expression changes in the high and low immune and stromal score groups, while heat maps were used to visualize DEGs.

### Enrichment analysis

The Database for Annotation, Visualization and Integrated Discovery (DAVID) was used to analyze enriched KEGG and GO terms for hub genes. GO analysis was based on the three root categories: biological process (BP), cellular component (CC), and molecular function (MF). According to the significance threshold of *P* < 0.05, bubble plots were generated to display the top five terms.

### Construction and verification of immune signatures

Immune signatures were first constructed by analyzing through univariate Cox regression those genes significantly related to survival in patients with TERTp mutations. By adding a penalty function (lambda), LASSO was used to eliminate redundant genes. Using the Akaike information criterion, multivariate Cox regression analysis was performed to develop a model for prognostic risk scoring. Risk scores were limited to 10 points. Kaplan-Meier survival analysis and ROC analysis were used to assess the prognostic accuracy of the risk model for glioma patients with mutant and wild-type TERTp.

### Immune cell analysis

The R package CIBERSORT was used to investigate the presence of 22 tumor-infiltrating immune cell types in TCGA-glioma cases with TERTp mutations. An unpaired *t*-test with significance set at *P* < 0.05 was used to compare immune cell distribution between high-risk and low-risk groups.

### Immunohistochemistry

From Guizhou Medical University Affiliated Hospital, 54 TERTp-mutant glioma tissues were collected prior to radiotherapy or chemotherapy, with approval from the Human Ethics Committee of Guizhou Medical University. All participants provided informed consent. The Human Research Ethics Review Committee of Guizhou Medical University approved the analysis of these clinical samples, which was carried out on basis of the tenets expressed in the Declaration of Helsinki. For immunohistochemistry (IHC), the sections were probed with the following antibodies: HOXC6 (1:200, ab41587, Abcam, Cambridge, UK), WT1 (1:100; 12609-1-AP, Proteintech, Wuhan, China), CD70 (1:500; 67749-1-Ig; Proteintech, Wuhan, China), and OTP (1:4000; ab254267; Abcam, Cambridge, UK).

### Immunotherapy response prediction and drug sensitivity analysis

The online tool TIDE (Tumor Immune Dysfunction and Exclusion) was used to predict potential ICB responses [35]. *In vivo* drug responses were predicted using OncoPredict, an algorithm developed by Maeser et al. [36]. In order to calculate the sensitivity to drugs of gliomas, OncoPredict scripts were used to match the gene expression matrix of each glioma sample to the antitumor effects of drugs in cancer cells recorded in the Cancer Cell Line Encyclopedia (Broad Institute, Cambridge, MA, USA). Patients with gliomas with high drug scores are less sensitive to anticancer drugs. The limma package was used to analyze differences in drug scores between patients at high and low risk, while |logFC| ≥1, and adjusted *P* < 0.05 were set as cut-offs for significance.

### Availability of data and materials

The datasets used and/or analyzed during the current study are available from the corresponding author on reasonable request.

## Supplementary Materials

Supplementary Table 1

Supplementary Table 2
