# Supplementary material for: Predicting the prognosis of glioma patients with TERT promoter mutations and guiding the specific immune profile of immune checkpoint blockade therapy
Source: Aging (Albany NY). 2024 Mar 18;16(6):5618–33. doi: 10.18632/aging.205668 (PMC11006486; doi:10.18632/aging.205668)
Supplement: Supplementary Table 2 [file aging-16-205668-s002.pdf]

SUPPLEMENTARY TABLES

Supplementary Table 2. The hazard rate of genes for glioma patients with TERT promoter-mutated.

ID	HR	HR.95L	HR.95H	*p*-value
IL10	1.401296707	1.16820136	1.680902392	2.78E-04
CD80	1.680121477	1.31873489	2.140542577	2.68E-05
CXCR3	1.673062748	1.311257916	2.134697472	3.48E-05
HOXA5	1.311948358	1.193513819	1.44213537	1.85892E-08
CD40LG	1.683021846	1.324759093	2.1381718	2.02E-05
IL2RA	1.263449436	1.085284275	1.470862993	2.57E-03
FCGR2A	1.570908668	1.242643354	1.985890831	1.59E-04
CD3D	1.416891407	1.188753932	1.688811456	0.000100121
HOXC6	1.339777582	1.191359826	1.506684992	1.05E-06
HAND2	1.308185324	1.170750093	1.46175418	2.10E-06
HOXA7	1.264175956	1.166146986	1.370445464	1.25E-08
HOXA10	1.263760788	1.165656311	1.370121977	1.36E-08
HOXB4	1.256479211	1.137275368	1.388177438	7.15E-06
KLRB1	1.317327982	1.082842817	1.602589947	5.86E-03
HOXA11	1.263335609	1.149044264	1.388995106	1.35E-06
HOXA4	1.253359993	1.154319813	1.36089778	7.57E-08
HOXA6	1.374021361	1.217284587	1.550939461	2.72E-07
HOXB5	1.402434859	1.199381587	1.639864707	2.25E-05
PTPN22	1.612489649	1.336986502	1.944763739	5.79E-07
CCL20	1.322543716	1.153477064	1.51639069	6.18E-05
CCR2	1.440138364	1.193514248	1.73772413	1.41E-04
EOMES	2.162706032	1.564438559	2.989760993	3.03E-06
HOXA3	1.300062396	1.187452601	1.423351325	1.37E-08
ICOS	1.8048284	1.412505119	2.306119469	2.34E-06
SHOX2	1.223249578	1.110456152	1.347499879	4.45E-05
AFP	1.562430931	1.248837288	1.954770599	9.46E-05
CD3G	1.891114604	1.449651399	2.467016861	2.63E-06
GATA4	1.307196099	1.145094066	1.492245652	7.32E-05
HLA-DQB2	1.333241464	1.123164091	1.582611851	1.01E-03
HOXA2	1.283245686	1.17444172	1.402129592	3.45E-08
HOXB6	1.278199257	1.080801373	1.511649949	4.13E-03
SLAMF1	1.817295749	1.404517107	2.351387407	5.52E-06
TREM1	1.262565437	1.127167166	1.414228103	5.62E-05
CLEC12A	1.341744641	1.140699448	1.578223506	3.86E-04
HOXA9	1.225208849	1.121304557	1.338741302	7.05E-06
HOXB2	1.297325529	1.169681194	1.438899367	8.40E-07
HOXD10	1.36713449	1.215792098	1.537316057	1.75E-07
NKX2-5	1.244500417	1.122792335	1.379401373	3.10E-05
WT1	1.300897745	1.158758861	1.460472062	8.35E-06
CD300LB	1.520548159	1.199903031	1.926877959	0.000524043
EMR1	1.426187179	1.192978147	1.704985021	9.74366E-05
GZMK	1.482573876	1.213433795	1.811409329	0.000116823
HOXC10	1.2943038	1.176692782	1.423670097	1.11153E-07
HOXC13	1.376912665	1.231280801	1.539769389	2.04992E-08

HOXC8	1.220296021	1.104497377	1.348235324	9.08623E-05
HOXC9	1.381213083	1.212463096	1.573449606	1.18776E-06
HOXD11	1.358327605	1.210728902	1.523919913	1.80763E-07
HOXD13	1.311717864	1.188299062	1.447955158	7.3699E-08
HOXB3	1.274676411	1.160273371	1.400359599	4.22954E-07
HOXB8	1.247854698	1.098546631	1.417455849	0.000660481
HOXC11	1.375777433	1.222296182	1.548531014	1.2504E-07
HOXD9	1.358154675	1.201031583	1.535833153	1.05995E-06
TRAT1	2.167431523	1.633716898	2.875503958	8.17295E-08
UBASH3A	1.817028631	1.388515496	2.377786246	1.35017E-05
CD70	1.642563121	1.332438182	2.024869628	3.3466E-06
CXCL6	1.257642315	1.101595506	1.435793976	0.000695177
FOXD3	1.208852224	1.014229337	1.440821761	0.034198992
IDO1	1.305190356	1.105804698	1.540526883	0.001638069
KRT7	1.320853258	1.135029896	1.537099011	0.000321571
PAX3	1.326831526	1.174648712	1.498730539	5.37224E-06
ZNF683	1.578651355	1.175757019	2.119604697	0.002389883
AREG	1.338353008	1.097220392	1.632478568	0.004036102
CEACAM4	1.341915123	1.027226522	1.753007889	0.031007269
CLEC5A	1.251684284	1.114414278	1.405862773	0.000152001
HOXA1	1.351151142	1.197873039	1.52404249	9.6411E-07
HOXA13	1.198591954	1.070500298	1.34201053	0.001681566
MMP7	1.198670225	1.054127433	1.363032839	0.005710016
OTP	1.552617782	1.345994136	1.790960238	1.56122E-09
POSTN	1.206300498	1.118697781	1.300763187	1.0832E-06
SAA1	1.190234398	1.100321451	1.287494596	1.38972E-05
SPAG17	1.370532233	1.187884542	1.581263613	1.56473E-05
TNFSF14	1.951613296	1.536187074	2.47938192	4.36635E-08
TREML2	1.556284703	1.200625914	2.017299517	0.000834258
